# Supplementary material for: Factors Associated with White Fat Browning: New Regulators of Lipid Metabolism
Source: Int J Mol Sci. 2022 Jul 11;23(14):7641. doi: 10.3390/ijms23147641 (PMC9325132; doi:10.3390/ijms23147641)
Supplement: Supplementary file 1 [file ijms-23-07641-s001.zip › ijms-1784185-supplementary.pdf]

**Supplement Table S1**

**Signal molecules and exogenous nutrients that regulate the browning of white fat**

| Classification | Name | Function | Mechanism of action | Origin | Year | References |
|---|---|---|---|---|---|---|
| Nutrients | Sesamol | Inhibit the increase in the number and quality of lipid droplets in brown adipose tissue, and promote the expression of brown fat marker genes | Inhibit the mRNA levels of white adipogenic genes PPARr, ACACA, SREBP-1c and FASN; promote the expression of brown fat marker genes such as UCP1, FGF21, COXII | Endogenous | 2020 | [62] |
| Nutrients | Resveratrol | Promote browning of white adipose tissue | Resveratrol activates SIRT1 to enhance the level of PPARγ deacetylation, reduces adipogenesis and lipid accumulation, and promotes the rate of lipolysis. SIRT1-mediated PPARγ deacetylation can promote the browning of white fat | Endogenous | 2014 | [75] |
| Nutrients | Ellagic acid | Inhibit the transformation of pre-adipocytes into mature adipocytes and promote the browning of white fat | Down-regulate the expression of PPARγ, C/EBPβ and C/EBPα in lipid synthesis-related proteins, and up-regulate the expression of BAT marker proteins UCP-1 and PGC-1a in WAT | Endogenous | 2019 | [78] |

| | | | | | | |
|---|---|---|---|---|---|---|
| Nutrients | Flavan 3 alcohol | The proliferation and differentiation of brown adipocytes and the related regulation process of white adipocyte browning that may be involved | After treatment with flavan-3 alcohol, the sympathetic nerve activity of mice increased, the content of no adrenaline increased, and the mRNA expression of UCP1 increased | Endogenous | 2018 | [82] |
| Nutrients | Epicatechin | Up-regulate the expression of brown adipose tissue to induce fat browning | Increase the expression level of PGC1α, TFAM, SIRT1, SIRT3, UCP1, etc. | Endogenous | 2017 | [84] |
| Nutrients | Capsaicin | Promote browning of white adipose tissue | The TRPV1 channel-dependent intracellular $Ca^{2+}$ increase and the phosphorylation of $Ca^{2+}$/calmodulin-activated protein kinase II and AMP-activated kinase promote Sirtuin-1 expression and activity, thereby triggering white adipocyte browning | Endogenous | 2016 | [86] |
| Nutrients | Curcumin | Induces browning of white adipocytes | Promote the expression of β3AR gene in inguinal white adipocytes, thereby increasing plasma norepinephrine levels; Secretion of interleukin (IL)-4 and/or IL-13 pathway to induce polarization of M2 macrophages and thus induce white adipocyte browning" | Endogenous | 2015;2016 | [90];[91] |

| | | | | | | |
|---|---|---|---|---|---|---|
| Nutrients | Berberine | Increase thermogenesis of brown fat cells and promote the browning of white fat cells | Berberine increases the expression of UCP1 and other thermogenic genes in white and BAT and primary adipocytes through mechanisms involving AMPK and PGC-1α | Endogenous | 2019 | [94] |
| Nutrients | Quercetin | Acts on fat cells and increases mitochondrial biogenesis to induce browning | Quercetin increased the expression of UCP1 and Elovl). The specific mechanism is reflected in the quercetin AMP-activated protein kinase (AMPK)/sirtuin1/PGC1α pathway | Endogenous | 2021 | [102] |
| Nutrients | Fucoxanthin | Improve insulin resistance and lower blood sugar levels, with potential anti-obesity effects | The intake of fucoxanthin increases the mRNA expression of β3 adrenergic receptor in WAT, which may be the reason for adaptive thermogenesis by stimulating the excitement of the sympathetic nervous system and up-regulating the expression of UCP1 | Endogenous | 2005 | [107] |
| Nutrients | Menthol | Enhance browning of WAT and improve diet-induced obesity | Menthol-induced TRPM8 activation, up-regulating the expression levels of UCP-1 and PGC-1α | Endogenous | 2017 | [110] |
| Nutrients | Chlorogenic acid | Induces browning of white adipocytes | Chlorogenic acid exerts anti-diabetic and anti-obesity effects through AMPK pathway, enhancing the expression of PPARγ, PRDM16 and PPGC-1α in brown and white adipose tissue | Endogenous | 2020 | [113] |

| | | | | | | |
|---|---|---|---|---|---|---|
| Nutrients | Chrysin | Reduce lipogenesis and lipogenesis, increase fat oxidation and induce browning | By AMPK activation, chrysin significantly up-regulates the expression of PGC-1α, UCP1, PRDM16, PPAR family and other browning genes in 3T3 preadipocytes; Stimulates the expression of PLIN, increases the anti-obesity effect of chrysin and promotes the recruitment of brown fat in white adipocytes | Endogenous | 2016 | [116] |
| Nutrients | Cinnamaldehyde | Inhibit the hypertrophy of adipose tissue and induce the browning of white adipose tissue | By enhancing the expression of UCP1 in brown adipose tissue and the expression of PPARγ, PRDM16 and PGC-1α proteins in brown and white adipose tissue | Endogenous | 2017 | [120] |
| Nutrients | Luteolin | Promote differentiation of primary brown cells and subcutaneous fat cells to brown | After luteolin treatment, the protein levels of UCP1, PGC1α and SIRT1 and the phosphorylation levels of AMPKα and ACC increased | Endogenous | 2016 | [122] |
| Nutrients | Taurine | Induces browning of white adipose tissue | Dependent on AMPK signal-mediated induction of PGC1mRNA in white adipocytes | Endogenous | 2019 | [124] |
| Nutrients | Emodin | Promote browning in scWAT and activate BAT activity | Increased beige adipocyte markers, such as mRNA in Cd137, Tmem26 and Tbx1scWAT, and UCP1, CD36, FATP4, PPARα, and inhibitory protein expression in scWAT and BAT | Endogenous | 2021 | [126] |

| | | | | | | |
|---|---|---|---|---|---|---|
| Nutrients | 3'-hydroxydaidzein | Induces browning of white adipose tissue | Increase PRDM16, C/EBP β, p-p38, SIRT1, PGC1 α and UCP1 protein expression | Endogenous | 2020 | [127] |
| Nutrients | Rice bran | Promote browning of white adipose tissue | Significantly up-regulate the expression of UCP1 protein and coding genes, and down-regulate the expression of TCF21 and HOXC8 (WAT-specific proteins). In addition, RRB, irb and irbs also effectively increased the levels of PRDM16 and PGC-1α | Endogenous | 2020 | [129] |
| Nutrients | Grape pomace extract | Promote the formation of brown-like fat cells | GPE prevents palmitate-mediated down-regulation of FNDC5/irisin protein expression and secretion through PGC-1α activation, and up-regulates the expression of UCP-1 in white adipose tissue (WAT) | Endogenous | 2020 | [131] |
| Nutrients | Purple Sweet Potato | Promote browning of white adipose tissue | Significantly up-regulate the expression of PGC1α and UCP-1 | Endogenous | 2021 | [132] |
| Nutrients | Dietary Apple Polyphenols | Promote browning of white adipose tissue | — | Endogenous | 2020 | [133] |
| Nutrients | Strawberry methanol extract | Promote browning of white adipose tissue | — | Endogenous | 2020 | [134] |
| Nutrients | Dietary silk peptide | Promote browning of white adipose tissue | — | Endogenous | 2020 | [135] |
| Nutrients | Lactobacillus amylophilus KU4 | Promote browning of white adipose tissue | — | Endogenous | 2019 | [136] |

| | | | | | | |
|---|---|---|---|---|---|---|
| Nutrients | Chitosan and Chitooligosaccharides | Promote browning of white adipose tissue | — | Endogenous | 2019 | [137] |
| Nutrients | Sargassum | Promote browning of white adipose tissue | — | Endogenous | 2020 | [138] |
| Nutrients | Freeze-dried Maqui (Aristotelia chilensis) berries | Promote browning of white adipose tissue | — | Endogenous | 2019 | [139] |
| Nutrients | Cardamom | Promote browning of white adipose tissue | — | Endogenous | 2019 | [140] |
| Nutrients | Standardized extract of psoralen seeds prenylated flavonoids | Promote browning of white adipose tissue | — | Endogenous | 2019 | [141] |
| Nutrients | Fermented Cordyceps Militaris Extract | Promote browning of white adipose tissue | — | Endogenous | 2019 | [142] |
| Nutrients | Genistein | Promote browning of white adipose tissue | — | Endogenous | 2019 | [143] |
| Nutrients | Broccoli | Promote browning of white adipose tissue | — | Endogenous | 2018 | [144] |
| Nutrients | Allicin | Promote browning of white adipose tissue | — | Endogenous | 2019 | [145] |

| | | | | | | |
|---|---|---|---|---|---|---|
| Signal molecule | Glucocorticoid | Inhibit the browning of white fat | GC can significantly down-regulate the level of UCP-1, but when the inhibitor RU486 is given, the expression level of brown functional genes such as UCP-1 increases significantly; GC regulates the expression of miR-27b through the GRE binding region of the upstream promoter region of miR-27b and inhibits the expression of its target gene PRDM16 | Exogenous | 2015 | [146] |
| Signal molecule | NRG4 | Has great potential in promoting the browning of white fat | The NRG4 synthesized in BAT is activated and phosphorylated under the combined action of protease and extracellular active protein fragments, and the expression of SREBP-1c is also significantly reduced. Changes in these factors reduce the transcription of ACC, SCD1 and FASN | Endogenous | 2018 | [150] |
| Signal molecule | Leptin | Participate in activating brown fat thermogenesis | Promote the expression of UCP1 and increase the mRNA level of UCP2 by 63% Sh2b1 neurons that mediate the sympathetic nervous system | Endogenous | 2001;2020 | [153];[154] |
| Signal molecule | Catecholamine | Promote browning of white adipose tissue | Catecholamines bind to G protein-coupled β-adrenergic receptors and activate adenylate cyclase (AC), which leads to an increase in cAMP to activate protein kinase A (PKA) and phosphorylate hormone- | Endogenous | 2017 | [156] |

| | | | | | | |
|---|---|---|---|---|---|---|
| | | | sensitive lipase (HSL). Causes the browning of white fat | | | |
| Signal molecule | FGF21 | Promote the browning of white adipose tissue; promote the recruitment of beige fat cells | FGF21 up-regulates the level of PGC-1α; at the same time, FGF21 acts in an autocrine/paracrine manner, thereby increasing the expression of UCP1 and other thermogenic genes in adipose tissue | Endogenous | 2017;2012 | [157];[48] |
| Signal molecule | BMP-9 | Convert white adipose tissue into brown adipose tissue; Promote the recruitment of beige fat cells | BMP-9 can transform white adipose tissue into brown adipose tissue by inhibiting liver gluconeogenesis; BMP-9 enhances the expression of fibroblast growth factor 21 and inhibits obesity, while FGF21 promotes the recruitment of beige adipocytes by up-regulating the protein expression of PGC1-α | Endogenous | 2020;2016 | [160];[161] |
| Signal molecule | Telmisartan | Induces browning of white adipocytes | Telmisartan induces M2 marker expression in a concentration-dependent manner in mouse macrophages, and PPARγ plays a key role in M2 polarization and white adipocyte browning; Telmisartan increases catecholamine (CA) and intracellular tyrosine hydroxylase (TH) mRNA levels | Endogenous | 2019 | [162] |

| | | | | | | |
|---|---|---|---|---|---|---|
| Signal molecule | IRISIN | Induces browning of white adipose tissue | Irisin circulating in the adipose tissue can induce the browning of white fat by activating the ERK and p38MAPK signaling pathways, thereby increasing the expression of UCP1 | Endogenous | 2014 | [60] |
| Signal molecule | Prostaglandin | Promote brown fat formation and white adipose tissue browning | Induced by cold exposure, it plays a key role in the formation of brown fat cells and white adipose tissue browning through the COX-2/PG pathway | Endogenous | 2018 | [165]. |
| Signal molecule | Atrial natriuretic peptide | Induces browning of white adipose tissue | By activating GC-A, it promotes the production of cGMP and then participates in the metabolic process. Through protein kinase G and ion channels, cGMP mediates the biological effects of ANP; Up-regulated the expression of UCP1 and other fat cell browning-related genes, increasing mitochondrial oxidative metabolism and fat oxidation | Endogenous | 2008;2014 | [168];[169] |
| Signal molecule | mTORC1 | Induces browning of white adipose tissue | β-adrenaline can stimulate the mTORC1/S6K1 pathway and activate the induced fat browning | Endogenous | 2016 | [171] |
| Signal molecule | PGC-lα | Promote the browning of white adipose tissue; activate thermogenesis of brown adipose tissue | Increase the expression of UCP1 and CIDEA; promote the division of mitochondria; increase the uncoupling activity of mitochondria | Endogenous | 2017;2011 | [41];[56] |

| | | | | | | |
|---|---|---|---|---|---|---|
| Signal molecule | Adrenaline | Activate brown fat | Increase the expression of genes related to thermogenesis and lipid metabolism; promote the accumulation of beige fat in subcutaneous fat | Endogenous | 2019 | [43] |
| Signal molecule | HDACl | The negative regulator of brown fat thermogenesis | Cold stimulation and B-adrenergic receptor activation can separate HDAC1 from the promoter region of brown fat-specific genes, thereby promoting the expression of brown fat-specific genes | Endogenous | 2016 | [51] |
| Signal molecule | JAK2 | | JAK2 and UCPl are both elevated in brown fat exposed to cold | Endogenous | 2013 | [52] |
| Signal molecule | ATF2 | Participate in the regulation of cold exposure on the adaptive thermogenesis of adipose tissue | — | Endogenous | 2020 | [53] |
| Signal molecule | Zfp516 | Participate in the regulation of cold exposure on the adaptive thermogenesis of adipose tissue | — | Endogenous | 2015 | [54] |
